# Summer crowds: An analysis of USFS campground reservations during the COVID-19 pandemic

**DOI:** 10.1371/journal.pone.0261833

**Published:** 2022-01-12

**Authors:** Mostafa Shartaj, Jordan F. Suter, Travis Warziniack

**Affiliations:** 1 Department of Agricultural and Resource Economics, Colorado State University, Fort Collins, Colorado, United States of America; 2 Rocky Mountain Research Station, United States Forest Service, Fort Collins, Colorado, United States of America; Universiti Teknologi Malaysia - Main Campus Skudai: Universiti Teknologi Malaysia, MALAYSIA

## Abstract

During the COVID-19 pandemic, US public land managers faced the challenge of catering to large increases in camping demand, while maintaining social distancing guidelines. In this paper, we use multivariate linear regression to analyze weekly changes in reservations to US Forest Service (USFS) campgrounds between 2019 and 2020. The regression models estimate the impact of local COVID infection rates, public health restrictions, and spatial spillovers from proximity to National Parks (NPs), metropolitan areas and wildfire on camping demand. Our sample includes 1,688 individual USFS campgrounds from across the contiguous US. The results illustrate the dramatic increases in camping on USFS land that occurred in the summer of 2020 and demonstrate that increases in local infection rates led to significant increases in camping nights reserved in the summer. The results also illustrate that the increase in camping nights reserved at USFS campgrounds was particularly dramatic for campgrounds located near large metropolitan areas and near NPs that saw increases in overall recreational visits. These results point to the important role that public lands played during the pandemic and can help guide public land resource allocations for campground maintenance and operation.

## Introduction

Over the last two decades, there has been a sustained increase in demand for outdoor recreation on public lands in the United States. At the same time, the budgets for operating and maintaining recreation sites on public lands have stagnated, resulting in widespread deferred maintenance backlogs [1, 2]. Within this context, the COVID-19 pandemic has precipitated an additional set of challenges for public land managers. Over the course of 2020, outdoor recreation emerged as one of the few accessible leisure activities generally deemed to be safe, and recent research points to increases in the values for nature-based recreation brought on by the pandemic [3]. Between March 1 and May 31 of 2020, the pandemic spurred mandatory stay-at-home orders for all residents in 36 states, with the remaining 14 states imposing orders for persons at increased risk or stay-at-home advisories [4]. In addition, many local, state, and national outdoor recreation sites were restricted or shuttered during the spring [5, 6]. The result was a documented decrease in household visits to public land recreation areas in the early stages of the pandemic [7]. The easing of public health restrictions in May and June of 2020, however, led to dramatic changes in recreation visits to public lands across the country. In this research, we document changes in demand for recreation on public land during the COVID-19 pandemic by analyzing data on reservations for USFS campgrounds.

The analysis makes use of a sample that includes weekly campground reservation data for 1,688 individual USFS campgrounds for 2019 and 2020. The rich dataset of campground reservations allows us to evaluate camping demand at a fine temporal scale for the entire contiguous US. We focus exclusively on USFS campgrounds to reduce the administrative heterogeneity that might exist if the analysis were to include campgrounds managed by other federal agencies (e.g., NPS, Bureau of Land Management) and because the USFS accounts for the vast majority of federally managed campgrounds that can be reserved. Conceptually, the focus of the analysis is to infer how demand for camping responded to direct and indirect COVID-19 induced changes experienced during 2020. Specifically, we employ a multivariate linear regression with weekly fixed effects to investigate how COVID-related variables impacted the change in weekly campground nights reserved between 2019 and 2020.

The first set of regression models that we estimate analyze the direct impact of changes in the local COVID infection rate and public health restrictions on camping demand. The dependent variable in these models is the change in weekly camping reservations between 2019 and 2020 for each campground in each week. By evaluating the change in reservations from one year to the next, the models control for time invariant characteristics of the campgrounds, like their size and location. We expect that higher local infection rates caused individuals to substitute away from leisure activities with a higher risk of infection, such as dining at restaurants, to leisure activities, such as camping, that allowed for more social distancing. A conceptual framework that illustrates how infection risk may cause individuals to substitute between leisure activities is provided in Section 1.1 in S1 Appendix. The models also evaluate how public stay-at-home orders and advisories, which varied across space over the course of 2020, impacted campground reservations.

The second set of models that we estimate add additional independent variables that account for the indirect impacts of COVID, which we label spatial spillovers, on camping demand. In particular, we investigate how proximity to urban areas and NP boundaries impacted the change in camping reservations in 2020 relative to 2019. Given the relative lack of leisure activities with adequate social distancing in cities, travel restrictions, and the large populations, we expect increases in camping demand in 2020 to be particularly large at campgrounds near urban areas. Similarly, since many campgrounds in NPs were closed or operating at reduced capacity in 2020, we expect USFS campgrounds near those NPs to see larger increases in demand, as households substituted USFS camping for NP camping. The regression models also control for occurrences of nearby wildfire activity, which we expect to depress camping demand.

This research makes three primary contributions. First, we show that following a decline in campground reservations in the spring of 2020, there was a dramatic increase in reservations in the summer months relative to the reservations made in 2019. Previous studies investigating the impact of COVID-19 induced changes on visitation to public lands have generally not included summer months and instead focused on the first COVID-19 wave [7–9]. As the largest proportion of individuals recreate during the summer, the studies miss out on the dramatic changes that occurred after the spring of 2020. A list of papers pertinent to the impacts of COVID-19 on outdoor recreation is provided in Section 1.2 in S1 Appendix for the interested reader. Second, we exploit local variation in COVID infection rates and public health policy directives over time and across space, to assess the impact they had on camping demand. Third, we illustrate how key spatial variables led to differential changes in demand for camping reservations. Craig [10], using a survey of 2,685 US respondents, reveals that individuals reported to be more likely to camp in closer proximity to their home due to COVID. We contribute to the literature by using reservations data to demonstrate that this preference for local camping led to the largest increases in reservations occurring near population centers. In addition, while most USFS campgrounds were fully open during the summer, many campgrounds in US NPs remained partially or fully closed. The resulting demand spillovers generated particularly large increases in reservations for USFS campsites in close proximity to NPs. There is a dearth of research that focuses on spillover effects of management decisions between the National Park Service (NPS) and the USFS. This study contributes to the literature by demonstrating spillover effects, which suggest the potential for benefits associated with a more coordinated response by the agencies. Overall, our results highlight the critical role that public lands played in providing recreational opportunities and a refuge from the challenges households faced during the pandemic year of 2020.

Understanding the impact of public policies, perceived risk of infection, and spatial demand spillovers can inform the USFS in understanding where they may beneficially allocate more resources. Moreover, many of the COVID-19 related impacts observed in 2020 are likely to persist, as the US struggles to keep COVID infections low. Over the course of the pandemic, markets for many goods and services, including protective equipment, medicine and hygiene products, have faced COVID-19 induced shortages [11, 12]. We add to the literature of COVID-19 induced impacts by demonstrating how the changes impacted camping in public lands.

This paper is organized into three major sections. The first section focuses on the direct impact of COVID-19 infection rates and public health restrictions on total nights reserved at USFS campgrounds. The second section demonstrates the indirect impacts of COVID by evaluating the role of spatial spillovers, controlling for infection rates and public health restrictions. Each of these sections begins with a data description, followed by model specification and results. The final section provides concluding thoughts and policy implications of this research.

## USFS reservations and COVID-19 infection rates

The empirical analysis evaluates the factors that impact reservations at USFS campgrounds in 2020 relative to 2019. Specifically, we use data on campground reservations made through recreation.gov in 2019 and 2020, made available by the USFS. The dataset has information on the final record of daily campground reservations, after excluding cancellations, voids and full refunds. In other words, in the data we observe only the camping reservations that were not canceled. We expect campsite reservations to be slightly different from actual visitation given that we do not observe whether individuals that reserved campsites actually showed up for the duration of their reservation. We also do not observe any walk-up camping that may have occurred from campers that did not make a reservation. Therefore, the results of the study reflect how COVID-19 induced changes impacted households’ intention to camp in 2020, relative to 2019.

The reservation data is combined with time invariant location data from the Recreation Information Database (RIDB) to get campground locations. The sample of campgrounds are limited to those in the contiguous United States. The sample of reservations only includes overnight camping reservations that do not include a discount pass, for which the total payment to reserve is greater than 0, have less then 11 occupants and the reservation length is between 0 and 11 days (exclusive). In general, campground reservations to USFS campgrounds can be made up to 6 months in advance. The analysis only includes reservations that have been reserved less than 201 days in advance. These filters help ensure that the analysis focuses on individual household campground visitation. After applying all filters, we retain 81.3 percent of all overnight reservations made to USFS campgrounds in the contiguous US.

For each campground we calculate the difference in total nights reserved for every week in 2020 compared to the same week in 2019. Only campgrounds with at least one reservation in both 2019 and 2020 are included in the analysis. This helps ensure a zero reservation in any week is not due to that campground no longer being operational. This criterion results in dropping less than 2 percent of reservations in 2019 and 2020. Thus, the exclusion is unlikely to impact our results. We then proceed with merging the data for weekly total nights reserved with data on campground capacity utilization, county-level infection rates, public health restrictions and the spatial variables of interest to create our final dataset. This process leads to 8 additional campgrounds dropping out. Thus, the final dataset includes total nights reserved at 1,688 campgrounds across the contiguous US, from week 7 to week 37 for both 2019 and 2020. To provide a reference, week 7 begins in early February and week 37 ends near mid-September. Campgrounds may have zero reservations during the off season for a given campground. To control for that, we exclude weeks for which a campground has zero reservations for both 2019 and 2020.

**Fig 1:**
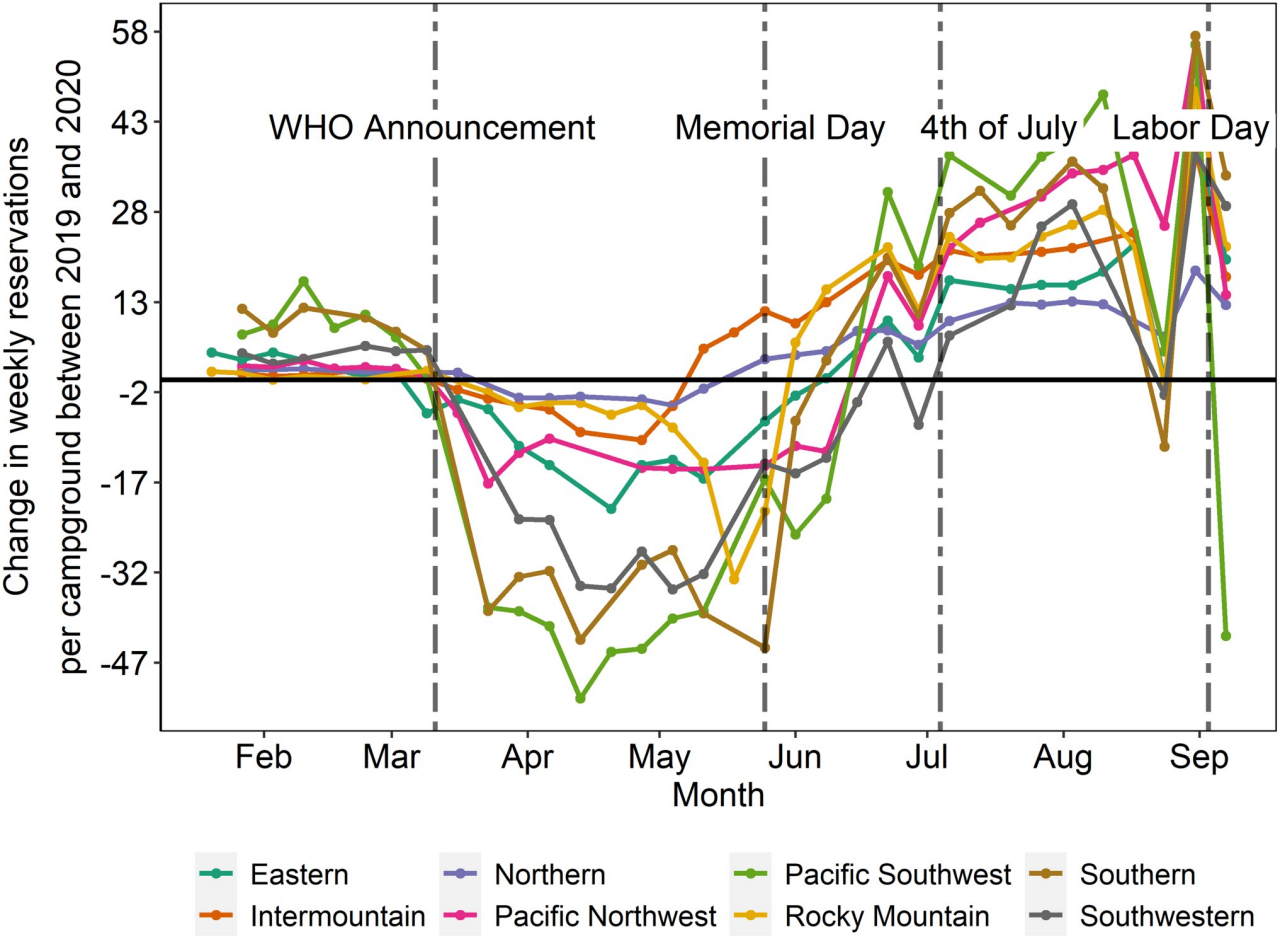
Change in weekly reservations per campground between 2020 and 2019 by USFS region.

Fig 1 shows the difference in reservations for the average campground by week in 2020 compared to 2019 by USFS region. USFS regions faced two distinct trends in the change in camping in 2020, relative to 2019. Following the WHO announcement of COVID-19 as a global pandemic on March 11 of 2020, we see a decline in USFS camping reservations compared to 2019. This decrease is likely driven by both policies at the state level and risk avoidance behavior by individuals. Then we see a gradual increase in relative camping nights reserved, starting from the week preceding Memorial day up until Labor day. In the analysis, summer is defined as the period between the week of Memorial day to the week of Labor day (inclusive). The dependent variable in the analysis is the change in weekly nights reserved between 2019 to 2020 for each campground.

The empirical analysis employs data on reservations and campground closures, provided by the USFS, which are used to estimate capacity utilization in 2019 and the number of days that a campground was closed in each week of 2020. Campgrounds that had high capacity utilization in 2019 have less availability to expand reservations, therefore we expect the 2019 capacity utilization to be negatively correlated with the change in camping reservations between 2019 and 2020. Daily capacity utilization is defined as the proportion of available campsites that are reserved on a given day at a given campground. We then calculate average capacity utilization for each campground for every week in 2019. Similarly, we calculate the daily proportion closed variable as the number of closed campsites divided by the number of campsites in inventory for each campground. Campgrounds only appeared in our closures data if they were available in the season. When a campground is off-season, we assume that all campsites for that specific campground were closed. If the proportion of closed sites for a given campground is greater than 0.85, then that campground is categorized as closed on that day. We then calculate the number of days each campground was closed for each week of 2020.

As shown in Table 1, the average number of nights reserved at a campground per week increased by nearly 28 percent in 2020. Additionally, there is a considerable decline in the average days in advance for each reservation and a slight decline in reservation length in 2020. Table 1 also reflects that the number of occupants per reservation and prices per night reserved remained more or less unchanged.

**Table 1:** Summary statistics for USFS campground reservations in 2019 and 2020.

Description	2019	2020	Difference	Percentage Change (%)
Mean days in advance	64.417	47.305	-17.112***	-26.564
Mean reservation length	3.283	3.179	-0.104***	-3.168
Mean number of occupants	3.967	3.971	0.004	0.101
Mean price per night	18.708	18.731	0.023	0.123
Mean nights reserved per week	39.251	50.346	11.095***	28.267

*Note:* The asterisks indicate p-values from unpaired t-tests on the difference in means;

*p<0.05;

**p<0.01;

***p<0.001.

The statistics presented in the table are estimated from the 1,688 campgrounds campgrounds in our sample.

### COVID infection rate and public policies

We use confirmed case data from USA Facts to calculate the county-level COVID-19 infection rate for every week [13, 14]. This dataset has been used in previous studies tracking and analyzing COVID-19 cases and deaths [15–17]. An increase in local COVID infection rates can result in higher perceived risk for all activities outside the home, which could lower demand for outdoor recreation. However, camping can be considered a relatively low risk activity compared to other forms of leisure, such as dining out. As a result, individuals may substitute to camping and away from riskier leisure activities as described in the conceptual model presented in Section 1.1 in S1 Appendix in Fig 8. Thus, how local infection rates impact camping demand becomes an empirical question. In this paper, the weekly infection rate is calculated as:

$$\begin{array}{c} r_{iw}=\frac{New\;Cases_{iw}}{Population_{i}}\times 100\;, \end{array}$$ (1)

where *r*_*iw*_ is the county-level infection rate at campground *i* in week *w*, *New Cases_iw_* is the number of new cases in week *w* in the county of campground *i* and *Population*_*i*_ is the population of the county of campground *i*. We assume that the county-level infection rate is not affected by the number of nights reserved at a campground. Given that camping is a relatively secluded form of recreation and documented infections lag exposure, it is reasonable to assume the nights reserved at an individual campground in a week do not have a significant impact on the county-level infection rate experienced in that week. The empirical model uses the 3-week moving average of the infection rate, which is calculated as:

$$\begin{array}{c} X_{1iw}=\frac{r_{iw}+r_{i(w-1)}+r_{i(w-2)}}{3}\;, \end{array}$$ (2)

where *X*_1*iw*_ is the 3-week moving average of the infection rate, and *r*_*i*(*w*−1)_ and *r*_*i*(*w*−2)_ are the lagged values of the infection rate. Along with the 3-week moving average, we have also tried modeling the infection rate with just the current and 2-week moving average infection rates. These results are presented in Table C2 of Section 1.4 in S1 Appendix. The results are not qualitatively different for these alternative specifications, therefore, we choose to focus on the 3-week moving average.

**Fig 2:**
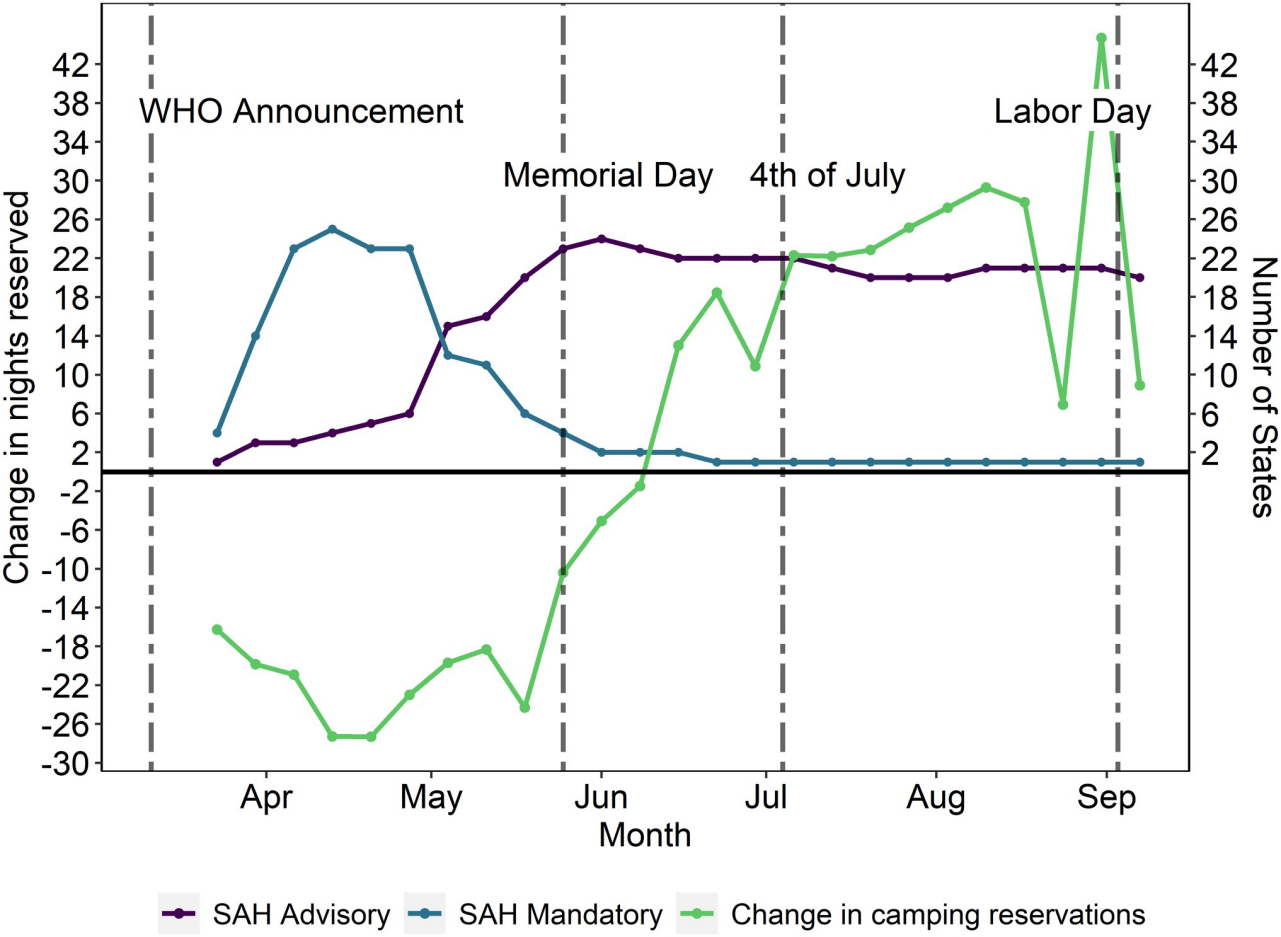
Average change in weekly camping nights reserved by campground between 2020 and 2019 and stay-at-home orders (SAH).

We also employ a dataset that provides county-level stay-at-home orders from the US Centers for Disease Control and Prevention (CDC). Fig 2 shows the number of states adopting different stay-at-home orders in weeks following the World Health Organization’s (WHO) announcement of COVID- 19 as a global pandemic. We see that a majority of the states issued mandatory stay-at-home orders, while fewer states issued less restrictive stay-at-home advisories. However, there is a relatively rapid decline in states keeping the mandatory stay-at-home orders in place in April and early May. Moreover, even among the states with mandatory stay-at-home orders, camping was still permitted in some states either initially or later on during the year. We also see an increase in the number of stay-at-home advisories as states reduced mandatory restrictions. The restrictions posed by stay-at-home advisories can vary by state but in general they are more lenient than mandatory stay-at-home orders.

The variation in policies enables us to identify how camping demand responded to mandatory stay-at-home orders or advisories, relative to having a more lenient or no stay-at-home order in place. In the majority of the cases, stay-at-home orders do not vary within a state. However, there are a few instances where counties might have a different stay-at-home policy than the one issued at the state level. We use county-level stay-at-home orders, whether issued by the county or by the state, to capture the impact of such heterogeneity. Partially restrictive orders, such as, stay-at-home orders for high risk individuals and only for certain areas, are less restrictive than mandatory stay-at-home orders for all individuals. While understanding the impact of these partial restrictions would be interesting, there is insufficient variation in the number of states that enacted these orders and therefore they are not included in the empirical analysis.

### Model specification: Infection rate and public health restrictions

The dependent variable in this paper represents the difference between total nights reserved for a specific week in 2020, relative to 2019, for each campground.

$$\begin{array}{c} \Delta Y_{iw}=Y_{iw2020}-Y_{iw2019}\;, \end{array}$$

where *Y*_*iw*_ is the the total nights reserved at campground *i* at week *w*. Camping demand and campground availability is seasonal. In order to ensure the change in camping reservations are comparable, we take the difference in total nights reserved on the same week across 2019 and 2020 for each campground. Differencing also helps remove potential time invariant unobservable characteristics at campgrounds that may confound our estimates. The regression model in this section is specified as

$$\begin{array}{c} \Delta Y_{iw}=\sum_{k=1}^{5}\beta_{k}X_{kiw}+\sum_{k=1}^{5}\gamma_{k}X_{kiw}S_{w}+\delta_{w}+\epsilon_{iw} \end{array}$$ (3)

where *δ*_*w*_ represents weekly fixed effects, which control for shocks common to all campgrounds over time and also help control for weeks that may be non-comparable across years. For instance, Labor Day in 2019 and 2020 fall in different weeks. *X*_1*iw*_ is the three week moving average of the county-level infection rate (including the current week), *X*_2*iw*_ is a dummy variable for mandatory stay-at-home orders, *X*_3*iw*_ is a dummy variable for stay-at-home advisory orders, *X*_4*iw*_ represents the number of days that campground *i* was closed in week *w* in 2020. Campgrounds already near full capacity in 2019 are less likely to experience substantial increases in reservations, compared to campgrounds that were not utilized as much. We therefore include the average capacity utilization in percentage points at the campground in 2019, labeled as *X*_5*iw*_. *S*_*w*_ is a dummy variable for summer that is interacted with each variable and *ϵ*_*iw*_ is the idiosyncratic error term. Interacting each variable with the summer dummy allows us to observe the heterogeneity in impacts of each independent variable between the spring and summer of 2020.

Table 2 presents an overview of the dependent and independent variables used in this study.

**Table 2:** Description of variables used in empirical analysis.

Short variable name	Notation	Description	Data source
Change in weekly camping nights reserved	Δ*Y*_*iw*_	Difference in nights reserved at campground *i* for week *w* between 2019 and 2020	Secondary data from the Recreation Information Database (RIDB) provided by the USFS
3-week moving avg infection rate	*X*_1*iw*_	3-week moving average infection rate experienced in the county of campground *i* in week *w*	Secondary data from USA Facts
Mandatory SAH	*X*_2*iw*_	Dummy variable for mandatory stay-at-home order in the county of campground *i* in week *w*	Secondary data from the US Center for Disease Control and Prevention (CDC)
Advisory SAH	*X*_3*iw*_	Dummy variable for advisory stay-at-home order in the county of campground *i* in week *w*	Secondary data from the US Center for Disease Control and Prevention (CDC)
Days Closed in 2020	*X*_4*iw*_	The number of days campground *i* was closed during week *w* in 2020	Secondary data provided by the USFS
Capacity utilization in 2019	*X*_5*iw*_	The 2019 percentage capacity utilization at campground *i* in week *w*	Secondary data provided by the USFS
Summer	*S*_*w*_	Dummy variable for summer weeks (22 ≤ *w* ≤ 37)	Determined as weeks between Memorial and Labor Day
Within 50 miles of NP	*X*_6*i*_	Dummy variable for campground *i* being within 50 miles of a NP boundary	Secondary data from Administrative Boundaries of National Park System dataset obtained from the NPS website
Increase in NP visits	*X*_7*iw*_	Dummy variable for campground *i* being within 50 miles of at least one NP that experienced an increase in recreational visits in week *w* in 2020, relative to 2019	Secondary data from NPS visitor use statistics on monthly recreational visits assigned to corresponding weeks
Small metro area	*X*_8*i*_	Dummy variable for campground *i* being within 10 miles of a small metropolitan area (population <250, 000)	Secondary data collected from the Rural Urban codes 2013 dataset from the Economic Research Service (ERS)
Medium metro area	*X*_9*i*_	Dummy variable for campground *i* being within 10 miles of a medium metropolitan area (250, 000≤ population <1, 000, 000)	Secondary data collected from the Rural Urban codes 2013 dataset from the ERS
Large metro area	*X*_10*i*_	Dummy variable for campground *i* being within 10 miles of a large metropolitan area (population ≥1, 000, 000)	Secondary data collected from the Rural Urban codes 2013 dataset made publicly available by the ERS
Wildfire boundary 10	*X*_11*iw*_	Dummy variable for campground *i* being within 10 miles of a wildfire boundary in week *w*	Secondary data from the 2020 Perimeters to Date dataset from the National Interagency Fire Center
Wildfire boundary 20	*X*_12*iw*_	Dummy variable for campground *i* being within 20 miles of a wildfire boundary in week *w*	Secondary data from the 2020 Perimeters to Date dataset from the National Interagency Fire Center

From Eq 3, we can derive the marginal effects for each variable, which can be represented as:

$$\begin{array}{c} \begin{array}{cc} \frac{\partial [\Delta Y_{iw}]}{\partial X_{kiw}} & =\{\begin{array}{cc} \beta_{k} & \text{if}\;\;S_{w}=0\\ \beta_{k}+\gamma_{k} & \text{if}\;\;S_{w}=1 \end{array} \end{array} \end{array}$$ (4)

For simplicity, Table 3 only reports the marginal effects demonstrated in Eq 4. The marginal effects when *S*_*w*_ = 0 are reported under Spring and the marginal effects when *S*_*w*_ = 1 are reported under Summer.

**Table 3:** Marginal effects from direct effects model.

	*Dependent Variable: Change in nights reserved*					
	(1)		(2)		(3)	
	Spring	Summer	Spring	Summer	Spring	Summer
3-week avg infection rate	-20.180	26.364*	-26.374	20.012^+^	32.401	24.726*
	(19.030)	(10.406)	(18.764)	(11.127)	(20.796)	(10.778)
Mandatory SAH	-16.420***	2.339	-18.538***	1.809	-4.006	1.788
	(3.024)	(2.574)	(3.023)	(2.703)	(4.972)	(3.883)
Advisory SAH	-9.096**	0.345	-10.920***	2.449	-12.388**	0.644
	(3.234)	(1.690)	(3.268)	(1.751)	(3.803)	(1.802)
Days closed in 2020	-1.813***	-6.311***			-1.329*	-6.058***
	(0.512)	(0.343)			(0.578)	(0.360)
Capacity utilization in 2019	-0.046^+^	-0.216***	-0.071*	-0.303***	-0.101**	-0.207***
	(0.027)	(0.022)	(0.030)	(0.023)	(0.032)	(0.024)
Observations	29,619		29,619		29,619	
Fixed effects	Weekly		Weekly		Region-by-week	
Clustered Standard Errors	Campground level		Campground level		Campground level	
R^2^	0.223		0.159		0.260	
Adjusted R^2^	0.222		0.157		0.253	
Residual Std. Error	44.936(df = 29578		46.753(df = 29580)		44.011(df = 29361)	
F statistic	211.9***(df = 40; 29578		146.7***(df = 38; 29580)		310.8***(df = 10; 29361)	

*Notes:*

^+^p<0.1;

*p<0.05;

**p<0.01;

***p<0.001 two-tailed;

The standard errors are clustered by campground and provided in parenthesis.

### Econometric results: Infection rate and public health restrictions

As shown in Table 3, specification 1 provides the marginal effects from Eq 4. Specification 2 is similar to 1 but excludes the number of days campgrounds were closed in 2020. Specification 3 is also similar to 1, but it uses a USFS region-by-week fixed effect instead of a weekly fixed effect. This is done to ensure that potential time variant changes due to differing USFS regional policies are controlled for. The full regression tables for each specification can be found in Tables B1-B3 in Section 1.3 of S1 Appendix. We see that the lagged 3-week average infection rate has robust signs across specifications for the summer. In the summer, an increase in the 3-week average infection rate led to between 24.73 to 26.36 more nights reserved per week at a typical campground in 2020, after controlling for the number of closed campground days. Given that the mean camping nights reserved in 2020 was 50.35, a 1 percentage point change in the 3-week moving average infection rate has a considerable impact on camping nights reserved. To put this in perspective, the mean 3-week avg infection rate in of the sample was 0.04 percentage points with a standard deviation of 0.067 percentage points. Thus, a 1 standard deviation increase in the infection rate led to an estimated 3.29 percent increase of the mean camping nights reserved in 2020. As the perceived risk of outside activity increased, the demand for less risky (more remote) outdoor activity led to increased camping demand. The magnitude of this coefficient decreases when we do not control for days closed. Counties that saw more infection rates are likely to have closed more of their sites, which decreased camping reservations. However, after controlling for the number of days a campground was closed, we see that the USFS campgrounds that remained open saw an increase in camping demand.

The mandatory SAH orders have a negative impact on the change in camping reservations in the spring weeks although the significance drops when the model includes region-by-week fixed effects. The mandatory SAH orders do not have a significant impact during the summer, although there were very few states that had mandatory SAH orders in the summer months, so the results are likely to be driven by only a few counties. The results for SAH advisory orders are similar, however the coefficient for the SAH advisory orders remains significant even after controlling for region-by-week differences for the spring weeks. The findings of camping demand being depressed in the Spring are in line with existing literature [7]. Not surprisingly, the number of days a campground is closed has a significant and negative impact on camping reservations. Additionally, looking at the capacity utilization of campgrounds in 2019, we see that campgrounds that had higher utilization rates for a given week in 2019 experience a significantly smaller change in camping reservations in 2020, as expected.

## Spillover effects: National parks, metros and wildfire

**Fig 3:**
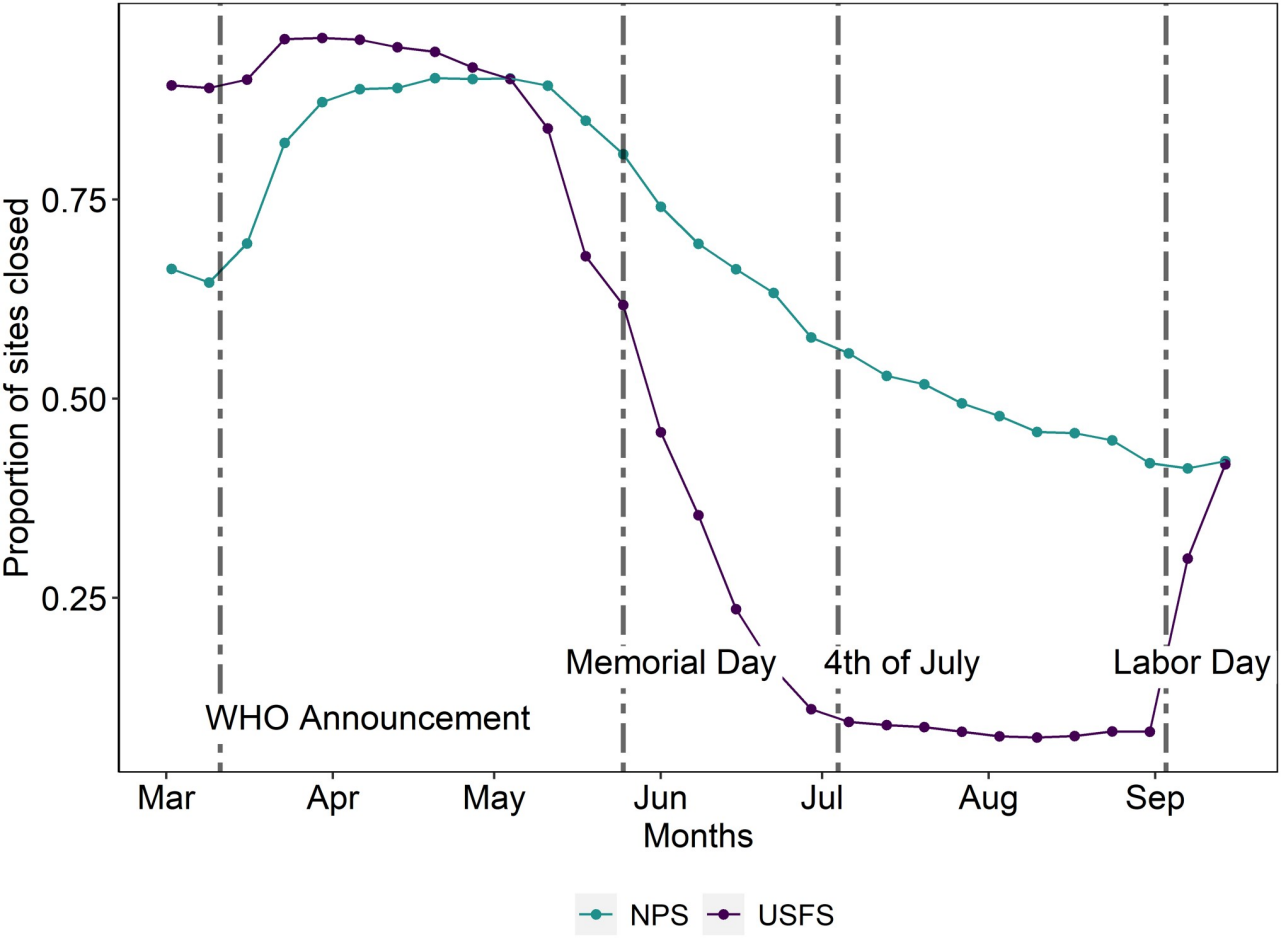
Proportion of USFS and NP campgrounds closed by week in 2020.

In this section, we explore how changes in campground nights reserved were impacted by a shift in risk attitudes and increased preferences for more remote activities during the COVID-19 pandemic. These spillovers represent the indirect impacts of COVID-19 induced changes on camping nights reserved. The spillover effects that we explore include spillovers from a campground’s proximity to (1) NPs; (2) metropolitan areas; and (3) wildfires. As shown in Fig 3, NP campgrounds experienced substantial closures throughout 2020, relative to USFS campgrounds. Despite the campground closures, most NPs remained opened for some level of day-use visitation. As a result, individuals who preferred to visit the NPs are likely to camp at nearby USFS campgrounds. Thus, we would expect the largest spillovers from NPs that experienced considerable recreation visits. The empirical model estimates this heterogeneous impact of NPs spillovers on USFS campground demand. Additionally, traveling during 2020 was either restricted or discouraged in most states. Furthermore, most metros also experienced non-essential business closures and provided reduced opportunities for other leisure activities. As a result, we expect to see the largest increases in reservations near high population metro areas in 2020, relative to 2019. Finally, we expect spillovers that reduce USFS camping demand nearby wildfire activity. Wildfires result in both increased risks and reduced air quality at campgrounds nearby. Given that many forests saw wildfire activity in 2020, we expect proximity to wildfire to reduce camping reservations in USFS campgrounds.

### National parks

**Fig 4:**
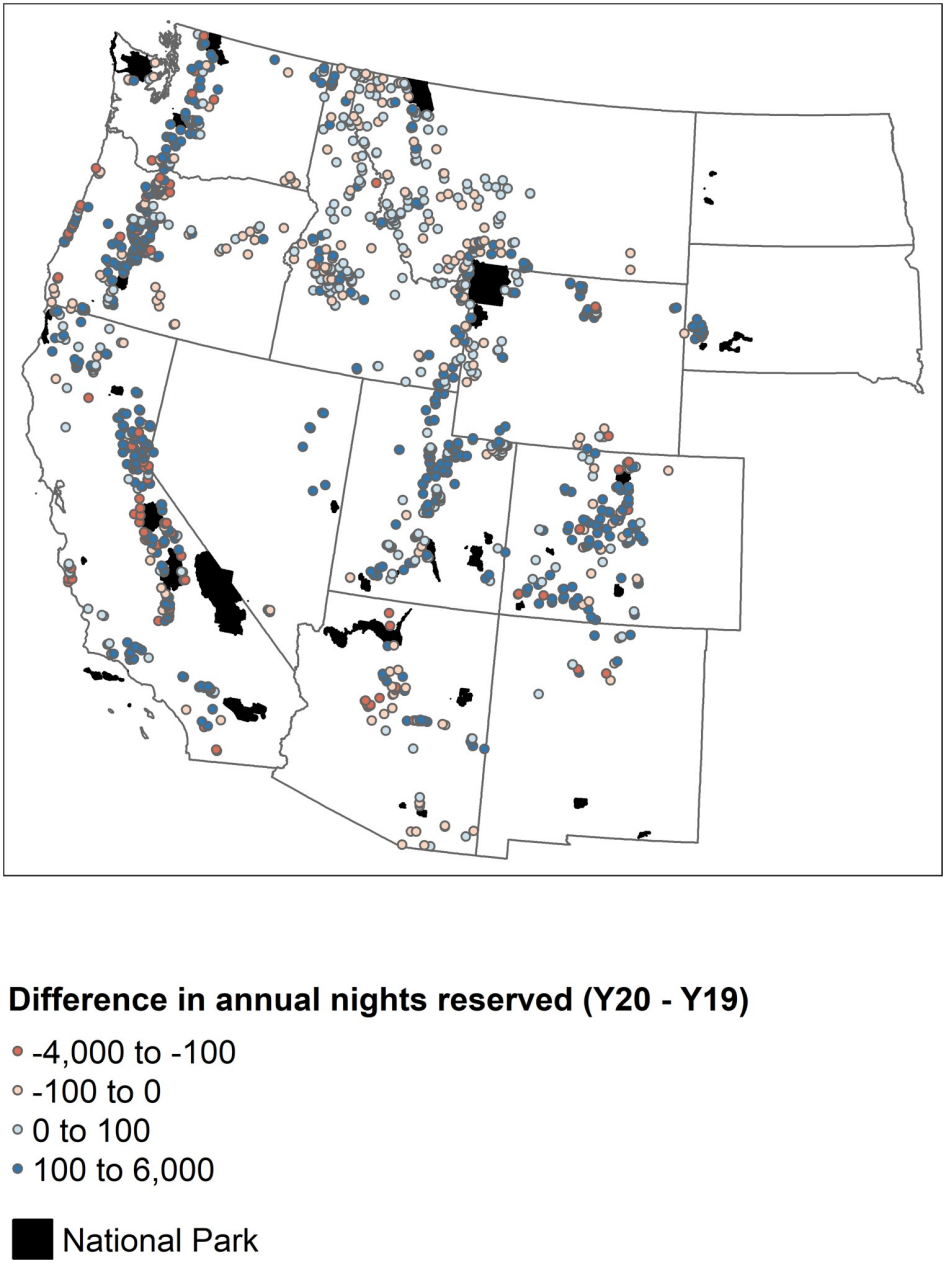
Difference in annual campground reservations between 2019 and 2020 at each campground.

We use NP boundary data from the NPS to identify USFS campgrounds near NPs. We also employ NPS visitor use statistics to estimate heterogeneous treatment effects of being near a NP [18]. We make use of campground closure data from recreation.gov to investigate the differences in closures between NP campgrounds and USFS campgrounds. Fig 4 displays the spatial distribution of campgrounds and NPs in the western US, as well as the total change in annual camping reservations between 2019 and 2020. The difference in annual nights reserved in Fig 4 is estimated by summing up total nights reserved from week 4 to week 37 for each year and then taking the difference. In order to identify potential spillover effects from NPs on nearby USFS campgrounds, we identify which USFS campgrounds fall within 50 miles of each NP. As initial campground locations are exogenous to changes experienced between 2019 and 2020, we are able to estimate the potential spillover effects from NPs on camping reservations in USFS campgrounds. We estimate the spillover effect on USFS camping reservations due to a campground being within 50 miles of a NP, relative to being further than 50 miles of a NP boundary.

**Fig 5:**
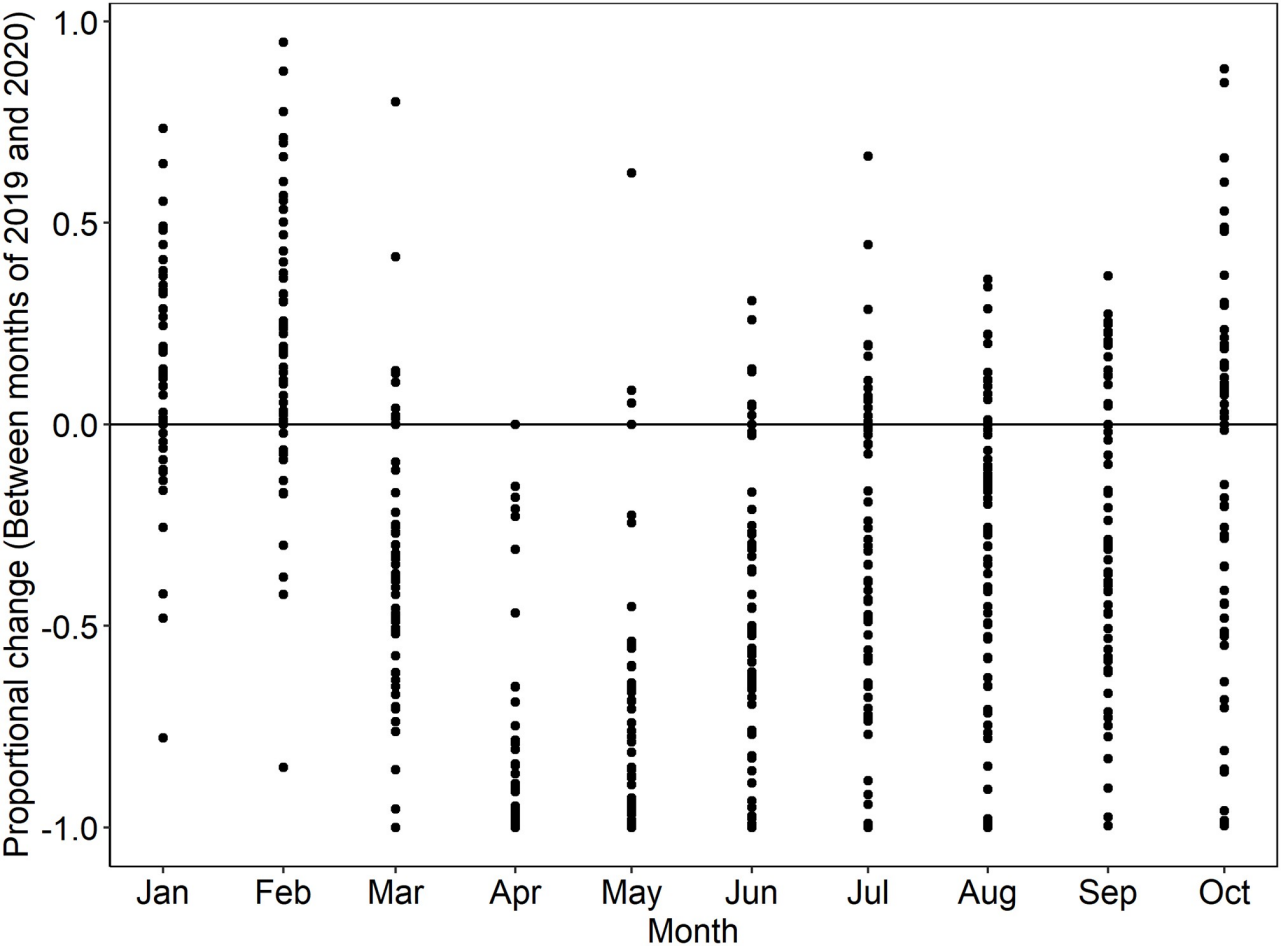
Proportional change in all recreation visits to NPs between 2019 and 2020.

We also explore the mechanism through which NP spillovers may impact nights reserved at USFS campgrounds. Individuals engaged in other forms of recreation at the NP may choose to camp at nearby USFS campgrounds. We demonstrate the impact of being near NPs as a function of the change in recreation visits to the NP between the months of 2019 and 2020. Each dot in Fig 5 represents the proportional change in NP visitation in a month of 2020, relative to 2019. Each dot represents a unique NP. As the figure shows, many NPs experienced large declines in visitation in March, April, and May. Some of the NPs experienced increased recreational visitation in the summer months in 2020 compared to 2019. We assign this monthly visitation data to the corresponding weeks and then assign dummy variables that indicate whether a USFS campground was near at least one NP, in a given week, that experienced an increase (above the zero line) in recreational visits in 2020.

### Metropolitan areas

**Fig 6:**
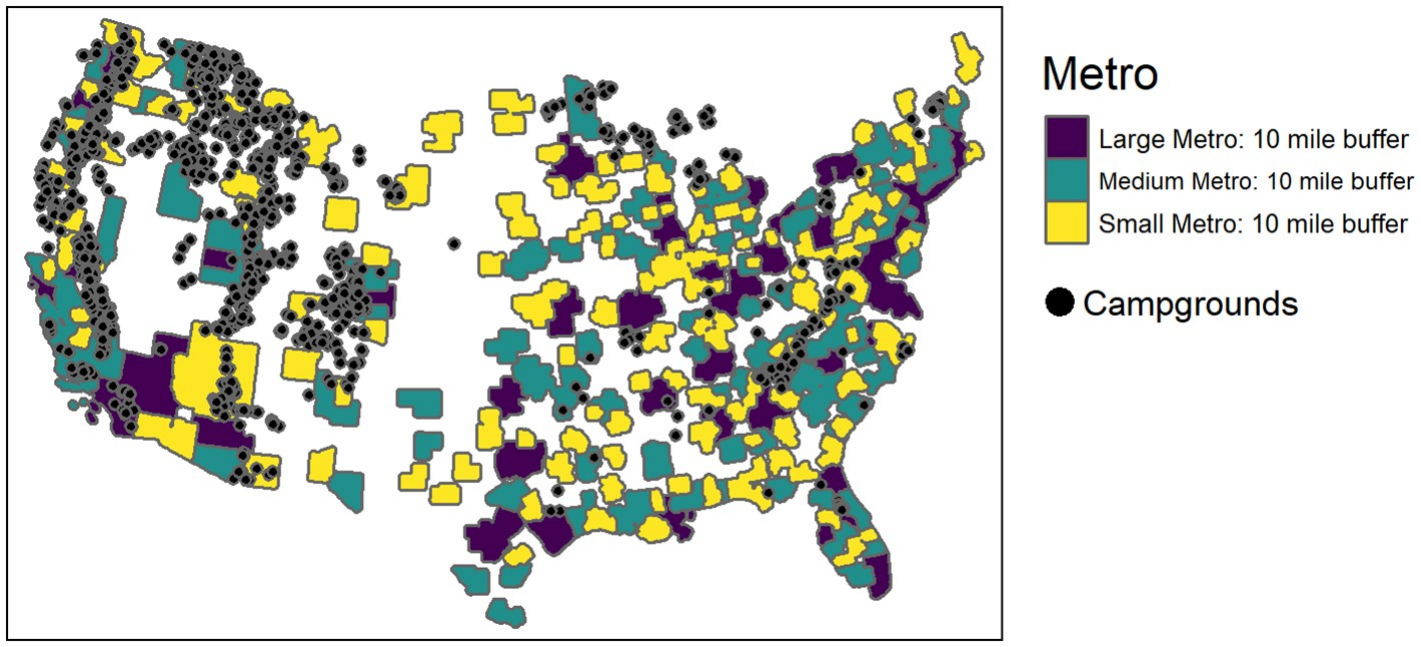
Map of metropolitan areas and campgrounds.

To evaluate the relationship between USFS reservations and proximity to population centers, we make use of data on the location of metropolitan counties using the Rural Urban codes 2013 dataset from the Economic Research Service (ERS). During 2020, most states had some form of travel restriction in place in an effort to reduce COVID transmission rates. Moreover, households were likely to avoid travel to reduce their risk exposure to the virus. Therefore, we expect increases in preferences for camping locally. The implication of an increase in demand for local camping is that we expect USFS campgrounds located near higher population density counties to experience a relative increase in reservations compared to campgrounds that are far from population centers. We investigate the extent to which changes in camping reservations were influenced by the campground location being within 10 miles of a metropolitan area boundary. We generate 10-mile buffers around the county shapes for each metropolitan area using R, with the “simple features” (sf) package. We then identify which campgrounds were located within these buffer areas. The metropolitan areas are categorized as metropolitan areas with less than 250,000 population, labeled as small metropolitan area, metropolitan areas with populations of 250,000 to 1 million, labeled medium metropolitan area, and finally metropolitan areas with more than 1 million population, labeled as large metropolitan area. These classifications are analogous to how the official Office of Management and Budget (OMB) defines metropolitan areas in the US. Fig 6 shows the geographical distribution of these metropolitan areas with a 10 mile buffer.

### Wildfire

**Fig 7:**
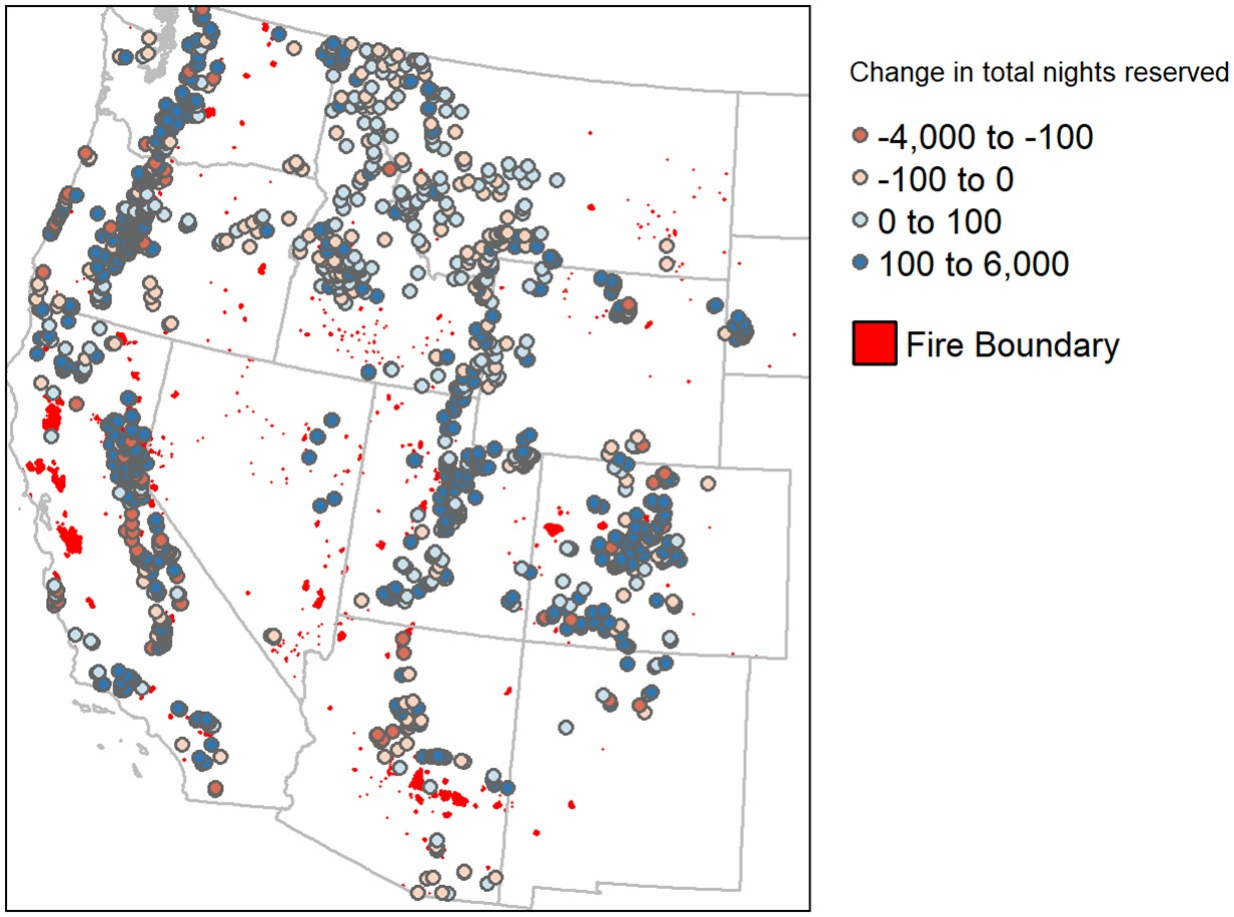
Wildfire perimeters in the western US in 2020.

In 2020, the US experienced considerable wildfire activity, with over 10 million acres burned according to National Interagency Fire Center (NIFC) estimates [19]. Existing research demonstrates that wildfire can result in a decrease in campground usage [20]. Wildfire also has the potential to cause NP closures and population movement that could influence COVID infection rates. This makes wildfire both an important control and a variable of interest, when looking at changes in camping demand in 2020. Fig 7 represents the wildfire boundaries experienced in the western US in 2020. To understand the impact of wildfire on demand for USFS campground reservations, we use the 2020 Perimeters to Date dataset from the NIFC [21]. It is important to note that some of the largest fires of 2020, including California’s August Fire, Arizona’s Cow Canyon Fire and Colorado’s Cameron Peak Fire, occurred after the study period. We also only look at fire activity within the contiguous US, due to focusing on camping only in those regions. In order to estimate the impact of wildfire on camping demand, we identify campgrounds located within 10 miles from a fire boundary and between 10 to 20 miles of a fire boundary in a given week. This does not count the small number of campgrounds within the fire boundary itself. Their inclusion does not meaningfully impact the results. These campground classifications are time variant due to fire boundaries changing over time. We expect campgrounds nearby fire boundaries to experience reduced reservations, due to increased risk and reduced air quality in those areas. The two distance buffers are included in the model to evaluate the extent to which the impact of wildfire on reservations dissipates with distance from the fire.

### Model specification: Spatial spillovers

The empirical model that we estimate to evaluate the impact of spillovers is specified as:

$$\begin{array}{c} \Delta Y_{iw}=\sum_{k=1}^{12}\alpha_{k}X_{kiw}+\sum_{k=1}^{12}\rho_{k}X_{kiw}S_{w}+\delta_{w}+\epsilon_{iw} \end{array}$$ (5)

where *δ*_*w*_ again represents weekly fixed effects and *ϵ*_*iw*_ is the idiosyncratic error term. The definitions of the dependent variable along with the independent variables *X*_1*iw*_, *X*_2*iw*_, *X*_3*iw*_, *X*_4*iw*_ and *X*_5*iw*_ are the same as that defined in Eq (3). *X*_6*i*_ is a time invariant dummy variable for campground *i* being in the 50 mile buffer of a NP boundary. We also test alternative specifications using different mutually exclusive buffer distances around NP boundaries. The results for these alternative specifications can be found in Table C1 of Section 1.4 in S1 Appendix and they show that alternative buffer distances do not qualitatively impact the results. The *X*_7*iw*_ variable is a dummy for campgrounds that fall within 50 miles of at least one NP that saw an increase in recreation visits in week *w* of 2020, relative to 2019. *X*_8*i*_, *X*_9*i*_ and *X*_10*i*_ represent dummy variables for campgrounds within 10 miles of small, moderate and large metropolitan areas respectively. *X*_11*iw*_ is a dummy variable that indicates if campground *i* was within 10 miles of the boundary of a wildfire in week *w*. *X*_12*iw*_ is a dummy variable that indicates if campground *i* was between 10 and 20 miles of a wildfire boundary. Similar to Eq (3), the model includes the summer dummy variable interacted with each independent variable.

From Eq 5, we can derive the marginal effects for each variable, which can be represented as:

$$\begin{array}{c} \begin{array}{cc} \frac{\partial [\Delta Y_{iw}]}{\partial X_{kiw}} & =\{\begin{array}{cc} \alpha_{k} & \text{if}\;\;S_{w}=0\\ \alpha_{k}+\rho_{k} & \text{if}\;\;S_{w}=1 \end{array} \end{array} \end{array}$$ (6)

Table 4 only reports the marginal effects demonstrated in Eq 6. The marginal effects when *S*_*w*_ = 0 are reported under Spring and the marginal effects when *S*_*w*_ = 1 are reported under Summer.

**Table 4:** Marginal effects for full model with spatial spillovers.

	*Dependent variable: Change in nights reserved*					
	(4)		(5)		(6)	
	Spring	Summer	Spring	Summer	Spring	Summer
3-week moving avg infection rate	-14.660	16.445	-19.230	11.096	33.878^+^	17.812^+^
	(17.754)	(10.363)	(17.360)	(11.059)	(19.988)	(10.532)
Mandatory SAH	-13.362***	-1.083	-15.272***	-1.420	-2.421	1.131
	(2.802)	(2.559)	(2.798)	(2.679)	(4.648)	(4.026)
Advisory SAH	-8.295*	-2.817^+^	-10.046**	-0.765	-11.262**	-2.417
	(3.306)	(1.664)	(3.333)	(1.706)	(4.011)	(1.809)
Within 50 miles of NP	-4.940^+^	1.695	-5.384*	1.773	-4.029	1.725
	(2.550)	(1.838)	(2.566)	(1.898)	(2.492)	(1.882)
Increase in NP visits	4.557	7.665**	5.294^+^	8.531**	4.971^+^	6.671**
	(2.826)	(2.528)	(2.834)	(2.610)	(2.568)	(2.472)
Small metropolitan area	-2.256	1.213	-2.938	-0.359	1.712	-1.303
	(2.150)	(1.812)	(2.151)	(1.904)	(2.277)	(2.079)
Medium metropolitan area	-10.580***	3.527	-10.759***	3.307	-4.676	2.489
	(3.121)	(2.169)	(3.131)	(2.278)	(3.387)	(2.338)
Large metropolitan area	-11.427**	15.700***	-11.857**	14.223***	-6.817	13.642***
	(4.149)	(2.888)	(4.194)	(3.056)	(4.424)	(3.119)
Wildfire 10	-9.178	-12.726***	-6.049	-17.663***	-25.539	-12.900***
	(16.006)	(2.779)	(15.366)	(3.092)	(16.398)	(2.820)
Wildfire 20	-15.461	-8.960***	-13.825	-8.383**	-25.749*	-9.650***
	(14.810)	(2.709)	(15.058)	(2.861)	(13.045)	(2.713)
Days closed in 2020	-1.709***	-6.336***			-1.354*	-6.001***
	(0.509)	(0.350)			(0.568)	(0.361)
Capacity utilization in 2019	-0.073*	-0.208***	-0.098**	-0.296***	-0.096**	-0.207***
	(0.030)	(0.021)	(0.032)	(0.022)	(0.031)	(0.023)
Observations	29,619		29,619		29,619	
Fixed effects	Weekly		Weekly		Weekly	
Clustered SEs	Campground level		Campground level		Campground level	
R^2^	0.239		0.175		0.270	
Adjusted R^2^	0.237		0.173		0.264	
Residual Std. Error	44.482(df = 29564)		46.315(df = 29566)		43.708(df = 29347)	
F statistic	177.1***(df = 24; 29564)		120.3***(df = 52; 29566)		40.13***(df = 271; 29347)	

*Note:*

^+^p<0.1;

*p<0.05;

**p<0.01;

***p<0.001 two tailed;

The standard errors are clustered by campground and provided in parentheses.

### Results: Spatial spillovers

The results from Table 4 for infection rates and mandatory stay-at-home orders are qualitatively similar to those observed in Table 3. The full regression results with interaction terms for Models 1, 2 and 3 of Table 4 are provided in Tables B4-B6, respectively, in Section 1.3 of S1 Appendix. The results show that having an advisory SAH, relative to having no SAH, resulted in reduced reservations for camping both in the summer and non-summer weeks after including the spatial variables as controls.

The results from Table 4 also reveal that USFS campgrounds near a NP that experienced increased visitation in 2020, relative to 2019, saw a considerable increase in reservations in the summer. The magnitude of the increase is approximately 13 percent of the mean value of campground nights reserved in 2020. The sign and significance of the effect is robust across all specifications. This result demonstrates a mechanism through which USFS campgrounds nearby NPs experienced spillovers during the pandemic. We see a reversal in the sign of the marginal effects for the Medium and Large metropolitan areas. Being near highly populated metros generally resulted in decreased camping in the spring. However, during the summer months campgrounds near populated metros experienced large increases in reservations, relative to other campgrounds. These marginal effects can be attributed to increased preferences for more remote activities, such as camping, amongst urban residents. The results illustrate that during the summer, USFS campgrounds within 10 miles of a large metropolitan area experienced around 13 to 15 more camping nights reserved, compared to campgrounds located further away. The magnitudes are of considerable size as they represent about 27 to 31 percent of the mean of camping nights reserved in 2020, respectively. The estimates from Table 4 also reflect that being near a fire boundary significantly reduced camping in the summer weeks. The magnitudes of the reductions are around 25 percent of the mean value of camping nights reserved in 2020. Finally, we see results similar to Table 3 for the campground closures and 2019 capacity utilization variables.

## Conclusion

In this research we demonstrate how COVID-19 infection rates, public health restrictions, and spillovers from proximity to NPs, metropolitan areas and wildfire, contributed to changes in USFS campground reservation demand in 2020, relative to 2019. A primary strength of the analysis is the use of national-level data on daily reservations at individual campgrounds. The results exemplify that increased local infection rates contributed to the increases in demand during 2020. As camping is a relatively socially distanced activity, it is sensible that camping was perceived as a preferable leisure activity when local infection rates were increasing. The findings also demonstrate that nearness to NPs and metropolitan areas resulted in increased demand, which can inform the USFS on where to expect large increases in visitation. The research also shows that proximity to NPs with increased recreational visits resulted in increased USFS campground reservations. The results are indicative of the fact that individuals that are recreating in a NP are likely to reserve USFS campgrounds nearby.

There are several policy implications based on the empirical results of the analysis. The first is that public health concerns and policies can drive rapid changes in recreation demand on public lands. In general, there is limited ability to quickly alter the infrastructure that supports public land recreation (e.g., campgrounds, trailheads, etc.). As a result, rapid changes in demand for recreational activities with particular attributes can create significant challenges for public land managers. Second, the results imply that USFS camping demand is impacted by changes in NP visitation. Thus, there is a potential benefit to coordination among agencies when facing national-level challenges. Third, the locational characteristics of particular campgrounds are critical for understanding the changes in demand that have occurred due to the pandemic. Campgrounds near urban areas and NPs have seen considerably larger increases in demand as a result of COVID-related factors. As recreation managers consider staffing decisions and investments in infrastructure, additional resources should be allocated to these campgrounds in close proximity to cities and NPs. Finally, it is likely that rapid increases in demand for campgrounds in 2020 caused many campgrounds to reach their capacity. This likely fueled an increase in dispersed camping, outside of established campgrounds, near these locations. Public land managers should consider the implications of these localized increases in dispersed camping on ecosystem health.

One weakness of the research is that we only observe campground reservations for overnight stays and thus we do not take into account walk up visitation to campgrounds. Given the increased uncertainty, whether walk up camping increased or decreased as a result of the pandemic remains an open empirical question. Additionally, the analysis only focuses on final reservations, as we only observe the record of the final disposition of the campground reservation. During times of increased demand, individuals may choose to reserve campgrounds ahead of time only to cancel them later on. Although, we see the mean days in advance reservations are made significantly decrease for the final reservations in 2020 (Table 1), it would be interesting to see whether that is due to a large number of cancellations made earlier.

Metropolitan areas generally faced greater COVID-related restrictions, which are the likely drivers for the increased reservations. However, we are unable to separate the heterogeneous impacts between population and specific restrictions, such as travel restrictions and business closures, on campground reservation demand. Additionally, we look at public health restrictions enacted largely at the state-level and do not investigate impacts of local policies, such as lockdowns. Recent research [22] points out that local policies to control infections, such as lockdowns, are often packaged with many other restrictions taking place at the same time. This makes it difficult to isolate the causal impact of the policy. Therefore, we focus on the impact of state-level packaged restrictions with varying degrees of leniency in this paper. Public health restrictions in one state are likely to impact reservations at out of state campgrounds that are relatively close to state borders. The potential impact of inter-state spillovers stemming from public health restrictions are not explored in this paper, but would make a fruitful area for future research.

## Supporting information

S1 Appendix (ZIP)

S1 Data (RAR)

## References

[1] Blahna DJ, Valenzuela F, Selin S, Cerveny LK, Schlafmann M, McCool SF. The shifting outdoor recreation paradigm: Time for change. In: Selin, Steven; Cerveny, Lee K; Blahna, Dale J; Miller, Anna B, eds 2020 Igniting research for outdoor recreation: linking science, policy, and action Gen Tech Rep PNW-GTR-987 Portland, OR: US Department of Agriculture, Forest Service, Pacific Northwest Research Station 257 p. 2020;987:9–22.

[2] Jenkins ME, Dougher C, Garlick C. Addressing the Maintenance Backlog on Federal Public Lands. 2020;.

[3] Morse JW, Gladkikh TM, Hackenburg DM, Gould RK. COVID-19 and human-nature relationships: Vermonters’ activities in nature and associated nonmaterial values during the pandemic. PloS one. 2020;15(12):e0243697. doi: 10.1371/journal.pone.0243697 33306716 PMC7732125

[4] Moreland A, Herlihy C, Tynan MA, Sunshine G, McCord RF, Hilton C, et al. Timing of state and territorial COVID-19 stay-at-home orders and changes in population movement—United States, March 1–May 31, 2020. Morbidity and Mortality Weekly Report. 2020;69(35):1198. doi: 10.15585/mmwr.mm6935a2 32881851 PMC7470456

[5] Kupfer JA, Li Z, Ning H, Huang X. Using Mobile Device Data to Track the Effects of the COVID-19 Pandemic on Spatiotemporal Patterns of National Park Visitation. Sustainability. 2021;13(16):9366. doi: 10.3390/su13169366

[6] Volenec ZM, Abraham JO, Becker AD, Dobson AP. Public parks and the pandemic: How park usage has been affected by COVID-19 policies. PloS one. 2021;16(5):e0251799. doi: 10.1371/journal.pone.0251799 34010353 PMC8133454

[7] Landry CE, Bergstrom J, Salazar J, Turner D. How Has the COVID-19 Pandemic Affected Outdoor Recreation in the US? A Revealed Preference Approach. Applied Economic Perspectives and Policy. 2020;.

[8] Rice WL, Pan B. Understanding drivers of change in park visitation during the COVID-19 pandemic: A spatial application of Big data. 2020;. 10.1016/j.wss.2021.100037 PMC8677329 34934999

[9] Geng DC, Innes J, Wu W, Wang G. Impacts of COVID-19 pandemic on urban park visitation: a global analysis. Journal of forestry research. 2021;32(2):553–567. doi: 10.1007/s11676-020-01249-w PMC7660132 33204057

[10] Craig CA. Camping, glamping, and coronavirus in the United States. Annals of Tourism Research. 2020;. 10.1016/j.annals.2020.103071 PMC7561296 33082612

[11] Ranney ML, Griffeth V, Jha AK. Critical supply shortages—the need for ventilators and personal protective equipment during the Covid-19 pandemic. New England Journal of Medicine. 2020;382(18):e41. doi: 10.1056/NEJMp2006141 32212516

[12] Romano S, Galante H, Figueira D, Mendes Z, Rodrigues AT. Time-trend analysis of medicine sales and shortages during COVID-19 outbreak: data from community pharmacies. Research in Social and Administrative Pharmacy. 2021;17(1):1876–1881. doi: 10.1016/j.sapharm.2020.05.024 32482587 PMC7245321

[13] USA FACTS. Detailed Methodology and Sources: COVID-19 Data; 2020. Available from: https://usafacts.org/articles/detailed-methodology-covid-19-data/.

[14] USA FACTS. US COVID-19 cases and deaths by state; 2021. Available from: https://usafacts.org/visualizations/coronavirus-covid-19-spread-map.

[15] Oster AM, Kang GJ, Cha AE, Beresovsky V, Rose CE, Rainisch G, et al. Trends in number and distribution of COVID-19 hotspot counties—United States, March 8–July 15, 2020. Morbidity and Mortality Weekly Report. 2020;69(33):1127. doi: 10.15585/mmwr.mm6933e2 32817606 PMC7439980

[16] Sarah HY, See I, Kent AG, Vlachos N, Whitworth JC, Xu K, et al. Characterization of COVID-19 in assisted living facilities—39 states, October 2020. Morbidity and Mortality Weekly Report. 2020;69(46):1730. doi: 10.15585/mmwr.mm6946a3 33211679 PMC7676639

[17] Andersen LM, Harden SR, Sugg MM, Runkle JD, Lundquist TE. Analyzing the spatial determinants of local Covid-19 transmission in the United States. Science of the Total Environment. 2021;754:142396. doi: 10.1016/j.scitotenv.2020.142396 33254938 PMC7498441

[18] National Park Service. Current Year Monthly and Annual Summary Report (1979—Present); 2020.

[19] National Interagency Fire Center. Wildfires and Acres; 2021. Available from: https://www.nifc.gov/fire-information/statistics/wildfires.

[20] Gellman J, Walls M, Wibbenmeyer MJ, et al. Wildfire, Smoke, and Outdoor Recreation in the Western United States. 2021;.

[21] National Interagency Fire Center. Wildland Fire Open Data; 2021. Available from: https://data-nifc.opendata.arcgis.com/.

[22] Goodman-Bacon A, Marcus J. Using Difference-in-Differences to Identify Causal Effects of COVID-19 Policies. In: Survey Research Methods. vol. 14; 2020. p. 153–158.

