# Supplementary material for: Summer crowds: An analysis of USFS campground reservations during the COVID-19 pandemic
Source: PLoS One. 2022 Jan 12;17(1):e0261833. doi: 10.1371/journal.pone.0261833 (PMC8754311; doi:10.1371/journal.pone.0261833)
Supplement: S1 Appendix — (ZIP) [file pone.0261833.s001.zip › S1 Appendix.pdf]

S1 Appendix

1.1 Conceptual Framework

In this section, we use a utility maximization framework to illustrate how the demand for one good (good B) changes in response to changes in the relative risk associated with another good (good A). Assume that goods A and B that are net substitutes. Good A requires individuals to participate in activities that might be associated with increased COVID infection risk, for example dining at a restaurant. If an individual is infected, they potentially incur health costs, hospital costs, and other costs due to physical and mental stress. Let the total cost of being infected be represented by i. Let π_A represent the probability of being infected when consuming A and π_B be the probability of being infected when consuming B. Let us also assume that $\pi_A > \pi_B$. Additionally, assume $\pi_B = 0$, that is individuals perceive B to have no infection risk. We can think of B representing goods such as camping that have minimal infection risk.

Let us assume individuals maximize utility from the consumption of these two goods. Let p_A and p_B represent prices for good A and B respectively, while X_A and X_B represents the units of consumption for each good. Let the income of the individual be represented by I and that consumers have the utility function, $U(X_A, X_B)$, which is quasi-concave, continuous, strictly increasing in X_A and X_B and differentiable. The consumer's probability of incurring cost $(p_A + i)$ per unit of good A is π_A and the probability of incurring a cost of p_A is $(1 - \pi_A)$. That is if infected, the consumer faces an additional cost of i along with the price of the good. This results in the consumer's expected budget constraint $I = \pi_A(p_A + i)X_A + (1 - \pi_A)p_A X_A + p_B X_B$, which simplifies to $I = (p_A + \pi_A i)X_A + p_B X_B$. Writing $\pi_A i = \theta_A$, the budget constraint becomes $I = (p_A + \theta_A)X_A + p_B X_B$, where θ is increasing in p_A and i. The consumer's utility maximization problem then becomes:

$$\begin{aligned} \underset{\{X_A, X_B\}}{\text{maximize}} \quad & U(X_A, X_B) \\ \text{subject to} \quad & I = (p_A + \theta_A)X_A + p_B X_B \end{aligned}$$

Given the assumptions, the demand functions derived from this optimization problem satisfy the general Slutsky equation [1]. The Slutsky equation for cross price effects for

good B can be written as:

$$\frac{\partial X_B}{\partial \theta_A} = \frac{\partial h_B}{\partial \theta_A} - X_A \frac{\partial X_B}{\partial I}, \tag{1}$$

where h_B is the Hicksian demand for good B. As good A and B are net substitutes $\frac{\partial h_B}{\partial \theta_A} > 0$. This term represents the substitution effect between the two goods. The second term represents the income effect. There is empirical support to suggest the relationship between camping demand and income is negative [2, 3]. We simply assume that the income effect is smaller than the substitution effect. Given these assumptions, we can see that $\frac{\partial X_B}{\partial \theta_A} > 0$. As θ_A is increasing in infection risk for good A, we can then deduce that increases in the perceived risk of good A will lead consumers to substitute to good B, causing an increase in the market demand for B. The market demand is simply the sum of all individual demand curves (represented in Fig 8 as D1). In the short run, we expect the supply and price of campsites within each campground to be fixed, which results in the campgrounds either facing excess demand or excess supply.

As the perceived risk of good A increases, the Marshallian demand for good B will shift outwards moving from D1 to D2, as shown in Fig 8. Campgrounds that are facing excess supply experience an increase in nights reserved from 2 to 4. For campgrounds facing excess demand, nights reserved would remain the same. This illustrates the importance of controlling for baseline capacity utilization. High capacity utilization campgrounds, represent sites that are already facing excess demand and thus have no room for growth, whereas, low capacity utilization campgrounds represent sites facing excess supply, and thus having room for experiencing growth in nights reserved.

Fig 8. Graphical exposition of changes in campground demand.

Infection risk can be moderated by the level of public health restrictions. Therefore, we control for public health restrictions in the analysis when estimating changes in the infection rates on camping reservation demand. This captures the direct impacts on camping nights reserved due to COVID-19.

1.2 Summary of literature on COVID-19 impacts on outdoor recreation

Table A1 provides a list of the limited literature that explores outdoor recreation impacts during the COVID-19 pandemic. The table includes brief summaries of each paper and elaborates how this paper builds on and contributes to the existing literature.

Table A1: Summary of pertinent literature of COVID-19 impacts on outdoor recreation.

Authors	Year	Paper title	Summary
Craig	2020	Camping, glamping and coronavirus in the United States.	Uses a survey of 2,685 US respondents to show that individuals had a higher preference for camping near their place of residence during the COVID-19 pandemic. We build on this literature by documenting the large increases in campground nights reserved at campgrounds near metropolitan areas.
Landry et al.	2020	How has COVID-19 pandemic affected outdoor recreation in the US? A revealed preference approach.	Uses a survey of 1,020 US respondents to collect information on individual trips to National Parks 12 months prior to the pandemic and 5 months into the pandemic. Their results document declines in camping in National Parks during the first few months of the pandemic. We contribute by documenting the increases in USFS campgrounds in the summer and by highlighting the determinants that lead to these increases.
Rice and Pan	2020	Understanding drivers of change in park visitation during the COVID-19 pandemic: A spatial application of Big data.	Pairs data on mobility trends provided by Google with data on public policies, county demographics, and spatial lattitude to demonstrate the importance of seasonality and median county age as contributing factors to outdoor recreation trends in the Spring of 2020. We contribute by demonstrating the changes in seasonal demand for camping reservations, by demonstrating the declines that occurred over the Spring, followed by increases in reservations in the Summer.
Geng et al.	2021	Impacts of COVID-19 pandemic on urban park visitation: a global analysis.	Uses Google's Community Mobility Reports to analyze trends in outdoor recreation around the world. The authors report an increase in the demand for outdoor green spaces that are a function of when outbreaks began in different countries.

Continued on next page

Table A1 – continued from previous page

Authors	Year	Paper title	Summary
Kupfer et al.	2021	Using mobile device data to track the effects of the COVID-19 pandemic on spatiotemporal patterns of National Park visitation.	Analysis uses spatiotemporal patterns in visitation to 5 NPs by combing visitation data with mobility records to show a decrease in visitation from March - May of 2020. As restrictions were reduced NP visitation in 2020 approached or surpassed 2019 levels. The authors show that the initial increases were from visitors coming from nearby states. Later in the year the visitors were came from a broader set of states. We contribute by analyzing reservations to USFS campgrounds, which make up the majority of campgrounds on public lands, to document nationwide impacts throughout 2020 and the role of spatial spillovers in boosting the reservations.
Volnec et al.	2021	Public Parks and the pandemic: How park usage has been affected by COVID-19 policies.	Use geo-tagged social media data from state, county and local parks in New Jersey to explore the impact of Covid-19 on park visitation and show that visitation increased by 63.4 percent at the beginning of the pandemic, then they dropped by 76.1 percent during park shutdown orders. Finally, they show that visitation returned to pre-shutdown levels once the parks in New Jersey reopened. We contribute to this literature by documenting nationwide changes in campground reservations. Additionally, we provide evidence on the mechanisms for the changes in reservations took to highlight the role of spatial spillovers.

1.3 Full regression tables

Table B1: Full regression with interaction terms for Model 1 of the infection rate section

	Dependent variable: Change in nights reserved
	Estimate [95 % CI low, 95 % CI high]
3-week moving avg infection rate	−20.180 [−57.477, 17.118]
	p = 0.289
3-week moving avg infection rate x Summer	46.544* [6.574, 86.513]
	p = 0.023
Mandatory SAH	−16.420*** [−22.347, −10.492]
	p = 0.00000
Mandatory SAH x Summer	18.759*** [11.734, 25.783]
	p = 0.00000
Advisory SAH	−9.096** [−15.434, −2.758]
	p = 0.005
Advisory SAH x Summer	9.441** []2.743, 16.140]
	p = 0.006
Days closed in 2020	−1.813*** [−2.817, −0.809]
	p = 0.0005
Days closed in 2020 x Summer	−4.499*** [−5.573, −3.425]
	p = 0.000
Capacity utilization in 2019	−0.046+ [−0.099, 0.007]
	p = 0.091
Capacity utilization in 2019 x Summer	−0.171*** [−0.233, −0.108]
	p = 0.00000
Observations	29,619
Fixed effects	Weekly
Clustered SEs	Campground level
R^2	0.223
Adjusted R^2	0.222
Residual Std. Error	44.936 (df = 29578)
F statistic	211.9*** (df = 40 ; 29578)
Note:	+p<0.10 ;*p<0.05; **p<0.01; ***p<0.001 two tailed;
	The standard errors used to estimate the confidence intervals are clustered by campground.

Table B2: Full regression with interaction terms for Model 2 of the infection rate section

	Dependent variable: Change in nights reserved
	Estimate [95 % CI low, 95 % CI high]
3-week moving avg infection rate	−26.374 [−63.151, 10.403]
	p = 0.160
3-week moving avg infection rate x Summer	46.386* [5.442, 87.329]
	p = 0.027
Mandatory SAH	−18.538*** [−24.463, −12.613]
	p = 0.000
Mandatory SAH x Summer	20.347*** [13.319, 27.375]
	p = 0.000
Advisory SAH	−10.920*** [−17.324, −4.516]
	p = 0.0008
Advisory SAH x Summer	13.369*** [6.574, 20.164]
	p = 0.0002
Capacity utilization in 2019	−0.071* [−0.129, −0.014]
	p = 0.016
Capacity utilization in 2019 x Summer	−0.232*** [−0.297, −0.167]
	p = 0.000
Observations	29,619
Fixed effects	Weekly
Clustered SEs	Campground level
R^2	0.159
Adjusted R^2	0.157
Residual Std. Error	46.753 (df = 29580)
F statistic	146.7*** (df = 38 ; 29580)
Note:	+p<0.10 ;*p<0.05; **p<0.01; ***p<0.001 two tailed; *The standard errors used to estimate the confidence intervals are clustered by campground.*

Table B3: Full regression with interaction terms for Model 3 of the infection rate section

	Dependent variable: Change in nights reserved
	Estimate [95 % CI low, 95 % CI high]
3-week moving avg infection rate	32.401 [−8.359, 73.161]
	p = 0.120
3-week moving avg infection rate x Summer	−7.675 [−52.418, 37.068]
	p = 0.737
Mandatory SAH	−4.006 [−13.751, 5.739]
	p = 0.421
Mandatory SAH x Summer	5.795 [−5.674, 17.263]
	p = 0.323
Advisory SAH	−12.388** [−19.841, −4.935]
	p = 0.002
Advisory SAH x Summer	13.032** [5.044, 21.020]
	p = 0.002
Days closed in 2020	−1.329* [−2.461, −0.197]
	p = 0.022
Days closed in 2020 x Summer	−4.729*** [−5.927, −3.531]
	p = 0.000
Capacity utilization in 2019	−0.101** [−0.163, −0.039]
	p = 0.002
Capacity utilization in 2019 x Summer	−0.106** [−0.180, −0.032]
	p = 0.005
Observations	29,619
R^2	0.260
Adjusted R^2	0.253
Residual Std. Error	44.011 (df = 29361)
F statistic	310.8*** (df = 10 ; 29361)
Note:	+p<0.10 ;*p<0.05; **p<0.01; ***p<0.001 two tailed; *The standard errors used to estimate the confidence intervals are clustered by campground.*

Table B4: Full regression with interaction terms for Model 1 of spatial spillovers section

	Dependent variable: Change in nights reserved
	Estimate [95 % CI low, 95 % CI high]
3-week moving avg infection rate	−14.660 [−49.458, 20.138]
	p = 0.409
3-week moving avg infection rate x Summer	31.105 [−6.188, 68.398]
	p = 0.103
Mandatory SAH	−13.362*** [−18.854, −7.869]
	p = 0.00001
Mandatory SAH x Summer	12.278*** [5.707, 18.849]
	p = 0.0003
Advisory SAH	−8.295* [−14.774, −1.815]
	p = 0.013
Advisory SAH x Summer	5.478 [−1.468, 12.423]
	p = 0.123
Within 50 miles of NP	−4.940+ [−9.938, 0.058]
	p = 0.053
Within 50 miles of NP x Summer	6.635* [0.722, 12.548]
	p = 0.028
Increase in NP visits	4.557 [−0.981, 10.095]
	p = 0.107
Increase in NP visits x Summer	3.108 [−3.380, 9.597]
	p = 0.348
Small metropolitan area	−2.256 [−6.470, 1.958]
	p = 0.295
Small metropolitan area x Summer	3.468 [−1.760, 8.696]
	p = 0.194
Medium metropolitan area	−10.580*** [−16.696, −4.464]
	p = 0.0006
Medium metropolitan area x Summer	14.107*** [6.565, 21.650]
	p = 0.0003
Large metropolitan area	−11.427** [−19.559, −3.295]
	p = 0.006
Large metropolitan area x Summer	27.127*** [17.347, 36.907]

Continued on next page

Table B4 – continued from previous page

	Dependent variable: Change in nights reserved
	Estimate [95 % CI low, 95 % CI high]
	p = 0.00000
Wildfire 10	−9.178 [−40.549, 22.192]
	p = 0.567
Wildfire 10 x Summer	−3.548 [−35.373, 28.277]
	p = 0.828
Wildfire 20	−15.461 [−44.488, 13.566]
	p = 0.297
Wildfire 20 x Summer	6.501 [−20.420, 33.421]
	p = 0.637
Days closed in 2020	−1.709*** [−2.706, −0.712]
	p = 0.00078
Days closed in 2020 x Summer	−4.628*** [−5.705, −3.550]
	p = 0.000
Capacity utilization in 2019	−0.073* [−0.133, −0.014]
	p = 0.016
Capacity utilization in 2019 x Summer	−0.135*** [−0.201, −0.068]
	p = 0.0001
Observations	29,619
Fixed effects	Weekly
Clustered SEs	Campground level
R^2	0.239
Adjusted R^2	0.237
Residual Std. Error	44.482 (df = 29564)
F statistic	177.1*** (df = 24 ; 29564)

Note: $^+$p<0.10 ;*p<0.05; **p<0.01; ***p<0.001 two tailed;
The standard errors used to estimate the confidence intervals are clustered by campground.

Table B5: Full regression with interaction terms for Model 1 of spatial spillovers section

	Dependent variable: Change in nights reserved
	Estimate [95 % CI low, 95 % CI high]
3-week moving avg infection rate	−19.230 [−53.254, 14.795]
	p = 0.268
3-week moving avg infection rate x Summer	30.326 [−7.721, 68.372]
	p = 0.119
Mandatory SAH	-15.272^{***} [−20.756, −9.788]
	p = 0.00000
Mandatory SAH x Summer	13.852^{***} [7.222, 20.482]
	p = 0.00005
Advisory SAH	-10.046^{**} [−16.578, −3.514]
	p = 0.003
Advisory SAH x Summer	9.281^{*} [2.253, 16.308]
	p = 0.010
Within 50 miles of NP	-5.384^{*} [−10.412, −0.355]
	p = 0.036
Within 50 miles of NP x Summer	7.157^{*} [1.096, 13.219]
	p = 0.021
Increase in NP visits	5.294^{+} [−0.260, 10.848]
	p = 0.062
Increase in NP visits x Summer	3.237 [−3.424, 9.897]
	p = 0.341
Small metropolitan area	−2.938 [−7.154, 1.279]
	p = 0.173
Small metropolitan area x Summer	2.579 [−2.748, 7.906]
	p = 0.343
Medium metropolitan area	-10.759^{***} [−16.896, −4.623]
	p = 0.00056
Medium metropolitan area x Summer	14.066^{***} [6.332, 21.800]
	p = 0.0004
Large metropolitan area	-11.857^{**} [−20.077, −3.637]
	p = 0.005
Large metropolitan area x Summer	26.080^{***} [15.833, 36.327]

Continued on next page

Table B5 – continued from previous page

	Dependent variable: Change in nights reserved
	Estimate [95 % CI low, 95 % CI high]
	p = 0.00000
Wildfire 10	−6.049 [−36.167, 24.068]
	p = 0.694
Wildfire 10 x Summer	−11.614 [−42.581, 19.353]
	p = 0.463
Wildfire 20	−13.825 [−43.338, 15.689]
	p = 0.359
Wildfire 20 x Summer	5.442 [−21.885, 32.769]
	p = 0.697
Capacity utilization in 2019	−0.098** [−0.161, −0.035]
	p = 0.003
Capacity utilization in 2019 x Summer	−0.198*** [−0.267, −0.128]
	p = 0.00000
Observations	29,619
Fixed effects	Weekly
Clustered SEs	Campground level
R^2	0.175
Adjusted R^2	0.173
Residual Std. Error	46.315 (df = 29566)
F statistic	120.3*** (df = 52 ; 29566)

Note: $^{+}$p<0.10 ;*p<0.05; **p<0.01; ***p<0.001 two tailed;
The standard errors used to estimate the confidence intervals are clustered by campground.

Table B6: Full regression with interaction terms for Model 1 of spatial spillovers section

	Dependent variable: Change in nights reserved
	Estimate [95 % CI low, 95 % CI high]
3-week moving avg infection rate	33.878^{+} [−5.297, 73.053]
	p = 0.091
3-week moving avg infection rate x Summer	−16.065 [−58.735, 26.604]
	p = 0.461
Mandatory SAH	−2.421 [−11.531, 6.690]
	p = 0.603
Mandatory SAH x Summer	3.552 [−7.862, 14.965]
	p = 0.542
Advisory SAH	-11.262^{**} [−19.123, −3.402]
	p = 0.005
Advisory SAH x Summer	8.845^{*} [0.270, 17.421]
	p = 0.044
Within 50 miles of NP	−4.029 [−8.912, 0.854]
	p = 0.106
Within 50 miles of NP x Summer	5.754^{+} [−0.134, 11.642]
	p = 0.056
Increase in NP visits	4.971^{+} [−0.061, 10.004]
	p = 0.053
Increase in NP visits x Summer	1.700 [−4.250, 7.650]
	p = 0.576
Small metropolitan area	1.712 [−2.751, 6.175]
	p = 0.453
Small metropolitan area x Summer	−3.016 [−8.770, 2.739]
	p = 0.305
Medium metropolitan area	−4.676 [−11.315, 1.962]
	p = 0.168
Medium metropolitan area x Summer	7.165^{+} [−0.703, 15.033]
	p = 0.075
Large metropolitan area	−6.817 [−15.488, 1.854]
	p = 0.124
Large metropolitan area x Summer	20.459^{***} [10.042, 30.876]

Continued on next page

Table B6 – continued from previous page

	Dependent variable: Change in nights reserved
	Estimate [95 % CI low, 95 % CI high]
	p = 0.0002
Wildfire 10	−25.539 [−57.679, 6.601]
	p = 0.120
Wildfire 10 x Summer	12.639 [−19.524, 44.802]
	p = 0.442
Wildfire 20	−25.749* [−51.317, −0.181]
	p = 0.049
Wildfire 20 x Summer	16.099 [−7.294, 39.491]
	p = 0.178
Days closed in 2020	−1.354* [−2.467, −0.240]
	p = 0.018
Days closed in 2020 x Summer	−4.647*** [−5.835, −3.459]
	p = 0.000
Capacity utilization in 2019	−0.096** [−0.156, −0.035]
	p = 0.003
Capacity utilization in 2019 x Summer	−0.112** [−0.183, −0.041]
	p = 0.003
Observations	29,619
Fixed effects	Region by week
Clustered SEs	Campground level
R^2	0.270
Adjusted R^2	0.264
Residual Std. Error	43.708 (df = 29347)
F statistic	40.13*** (df = 271 ; 29347)
Note:	+p<0.10 ;*p<0.05; **p<0.01; ***p<0.001 two tailed;
	The standard errors used to estimate the confidence intervals are clustered by campground.

1.4 Robustness tests

The majority of USFS campgrounds are located in the western US, therefore we estimate the model described in equation (4), but with only including campgrounds

from western states. The results are presented in specification (1) of Table C1. We see the results are similar to previous estimates using the entire sample. In specification (2), we estimate the model described in equation (4) but exclude the increase in NP visits variable and instead include two alternative distances. The variables 'Within 75 of NP' and 'Within 100 of NP' represent dummy variables for campgrounds between 50 to 75 miles and between 75 to 100 miles of a NP, respectively. We see that campgrounds that lie within 50 miles of a NP experienced increases in campground reservations in 2020, relative to campgrounds further away. Moreover, the magnitude and significance falls as we include campgrounds that lie further than 50 miles of a NP, as represented by the coefficient estimates of 'Within 50 miles of NP' and 'Within 100 miles of NP'.

Table C1: Marginal effects for alternative models.

	Dependent variable: Change in nights reserved			
	(1)		(2)	
	Spring	Summer	Spring	Summer
3-week moving moving avg infection rate	-50.703*	20.103+	-12.000	16.243
	(22.097)	(11.151)	(17.780)	(10.370)
Mandatory SAH	-18.608***	-2.249	-13.347***	-1.865
	(3.290)	(2.706)	(2.798)	(2.640)
Advisory SAH	-8.866**	-2.465	-8.245*	-3.114+
	(2.827)	(1.763)	(3.350)	(1.704)
Within 50 miles of NP	-3.977	1.515	-4.176	4.783*
	(2.420)	(1.908)	(2.598)	(2.122)
Within 75 miles of NP			-1.978	1.139
			(3.085)	(2.162)
Within 100 miles of NP			1.891	-0.598
			(2.638)	(2.380)
Increase in NP visits	4.944+	8.228**		
	(2.695)	(2.541)		
Small metropolitan area	-2.496	1.725	-2.557	1.091
	(2.104)	(1.952)	(2.121)	(1.814)
Medium metropolitan area	-6.541*	5.027*	-10.950***	3.597
	(3.021)	(2.365)	(3.158)	(2.184)
Large metropolitan area	-15.059***	15.572***	-11.480**	15.535***
	(3.967)	(2.994)	(4.135)	(2.920)
Wildfire 10	-14.238	-12.210***	-9.498	-13.092***
	(16.627)	(2.782)	(15.981)	(2.817)
Wildfire 20	-19.034	-9.197***	-15.722	-8.994**
	(14.395)	(2.729)	(14.758)	(2.734)
Days closed in 2020	-1.229**	-6.700***	-1.755***	-6.351***
	(0.473)	(0.395)	(0.512)	(0.350)
Capacity utilization in 2019	-0.075*	-0.230***	-0.070*	-0.208***
	(0.032)	(0.025)	(0.030)	(0.021)
West only	Yes		No	
Observations	24,469		29,619	
Fixed effects	Weekly		Weekly	
Clustered SEs	Campground level		Campground level	
R^2	0.243		0.237	
Adjusted R^2	0.242		0.236	
Residual Std. Error	44.19(df = 24414)		44.53(df = 29562)	
F statistic	145.5***(df = 54; 24414)		164.2***(df = 56; 29562)	
Note:	+p<0.1; *p<0.05; **p<0.01; ***p<0.001 two-tailed; *The standard errors are clustered by campground and provided in parentheses.*			

Table C2 presents the impact of alternative definitions used for the infection rate. The marginal effects from the table reflect that the sign and significance of the infection rate is robust to the different definitions used. Additionally, increasing the average number of weeks in the infection rate slightly increases both the magnitude of the marginal effect and the standard error.

Table C2: Marginal effects for alternative infection rate definitions.

	Dependent variable: Change in nights reserved			
	(1)		(2)	
	Spring	Summer	Spring	Summer
Weekly avg infection rate	0.470	13.444^{+}		
	13.944	8.161		
2-week avg infection rate			-9.367	15.747^{+}
			15.963	9.558
Mandatory SAH	-13.426^{***}	-1.055	-13.433^{***}	-1.081
	2.799	2.558	2.803	2.557
Advisory SAH	-8.013^{*}	-2.843^{+}	-8.227^{*}	-2.822^{+}
	3.290	1.663	3.303	1.664
Within 50 miles of NP	-4.950^{*}	1.788	-4.899^{+}	1.726
	2.460	1.826	2.524	1.833
Increase in NP visits	5.015^{+}	7.574^{**}	4.655	7.613^{**}
	2.804	2.527	2.869	2.527
Small metropolitan area	-2.132	1.252	-2.182	1.224
	2.024	1.809	2.088	1.811
Medium metropolitan area	-9.469^{**}	3.548	-10.001^{***}	3.523
	2.904	2.175	3.009	2.172
Large metropolitan area	-10.272^{**}	15.739^{***}	-10.919^{**}	15.708^{***}
	3.894	2.889	4.037	2.888
Wildfire 10	-9.233	-12.687^{***}	-9.222	-12.699^{***}
	16.024	2.776	16.017	2.778
Wildfire 20	-15.965	-8.895^{**}	-15.694	-8.914^{***}
	14.771	2.709	14.790	2.708
Days closed in 2020	-1.693^{***}	-6.332^{***}	-1.703^{***}	-6.335^{***}
	0.502	0.350	0.505	0.350
Capacity utilization in 2019	-0.071^{*}	-0.208^{***}	-0.071^{*}	-0.208^{***}
	0.028	0.021	0.029	0.021
Observations	30,007		29,814	
Fixed effects	Weekly		Weekly	
Clustered SEs	Campground level		Campground level	
R^2	0.238		0.239	
Adjusted R^2	0.237		0.237	
Residual Std. Error	44.22(df = 29950)		44.35(df = 29758)	
F statistic	167.5^{***}(df = 56; 29950)		169.6^{***}(df = 55; 29758)	

Note: $^{+}$p<0.1; *p<0.05; **p<0.01;***p<0.001 two-tailed;
The standard errors are clustered by campground and provided in parentheses.

References

1. Jehle G, Reny P. Advanced Microeconomic Theory (Third)'. Essex: Pearson Education Limited. 2011;.

2. Brox JA, Kumar RC. Valuing campsite characteristics: A generalized travel-cost model of demand for recreational camping. Environmetrics: The official journal of the International Environmetrics Society. 1997;8(2):87–106.

3. Shartaj M, Suter JF. Exploring the Local Determinants of Campground Utilization on National Forest Land. In: Western Economics Forum. vol. 18; 2020. p. 114–128.
