# Supplementary figures and images for: Summer crowds: An analysis of USFS campground reservations during the COVID-19 pandemic
Source: PLoS One. 2022 Jan 12;17(1):e0261833. doi: 10.1371/journal.pone.0261833 (PMC8754311; doi:10.1371/journal.pone.0261833)

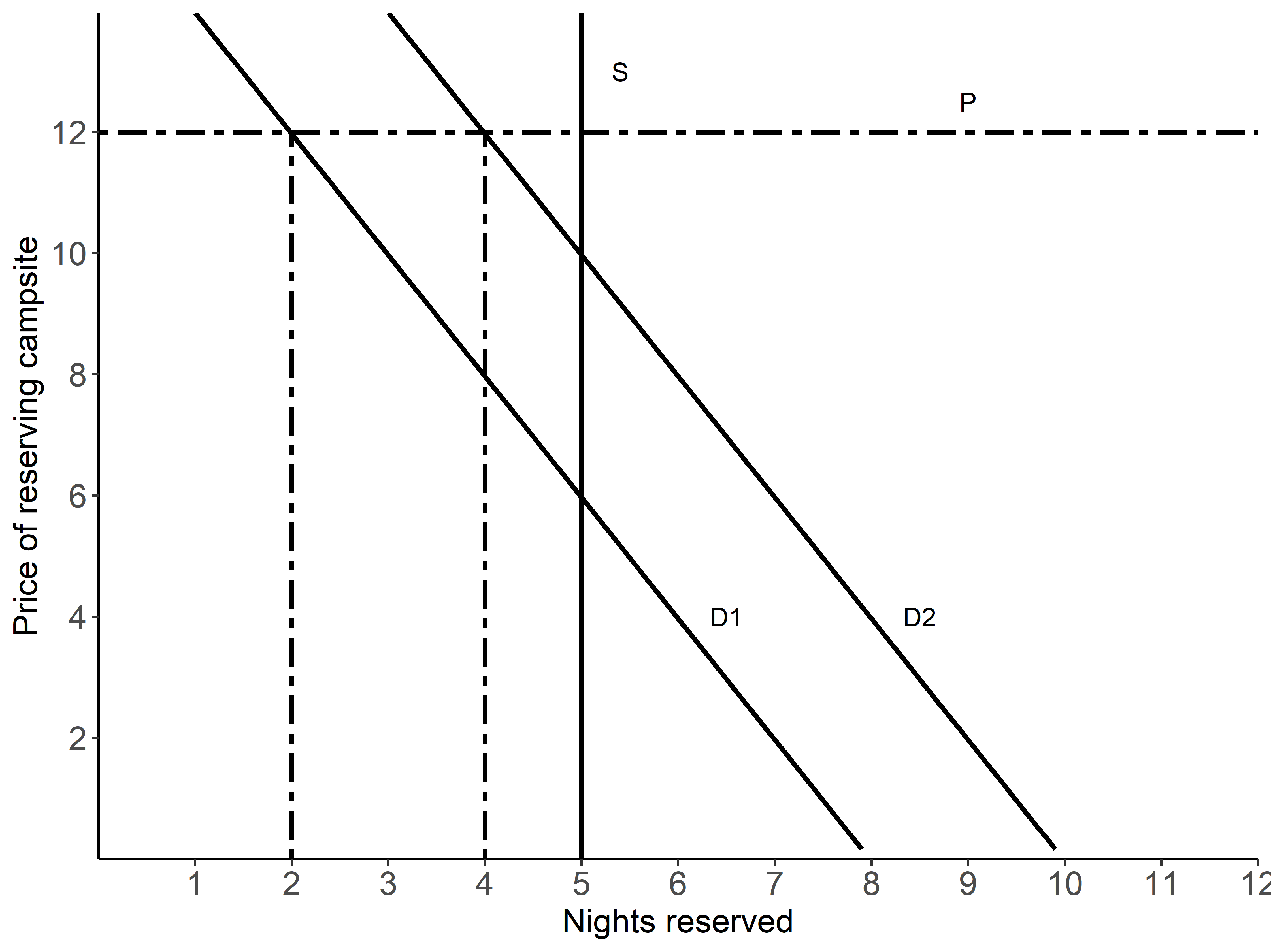

Supplement: S1 Appendix — (ZIP) [file pone.0261833.s001.zip › Fig8.tif]
